# Supplementary material for: Cryo-EM structures of orphan GPR21 signaling complexes
Source: Nat Commun. 2023 Jan 13;14:216. doi: 10.1038/s41467-023-35882-w (PMC9839698; doi:10.1038/s41467-023-35882-w)
Supplement: Supplementary file 1 — Supplementary Information [file 41467_2023_35882_MOESM1_ESM.pdf]

Supplementary information

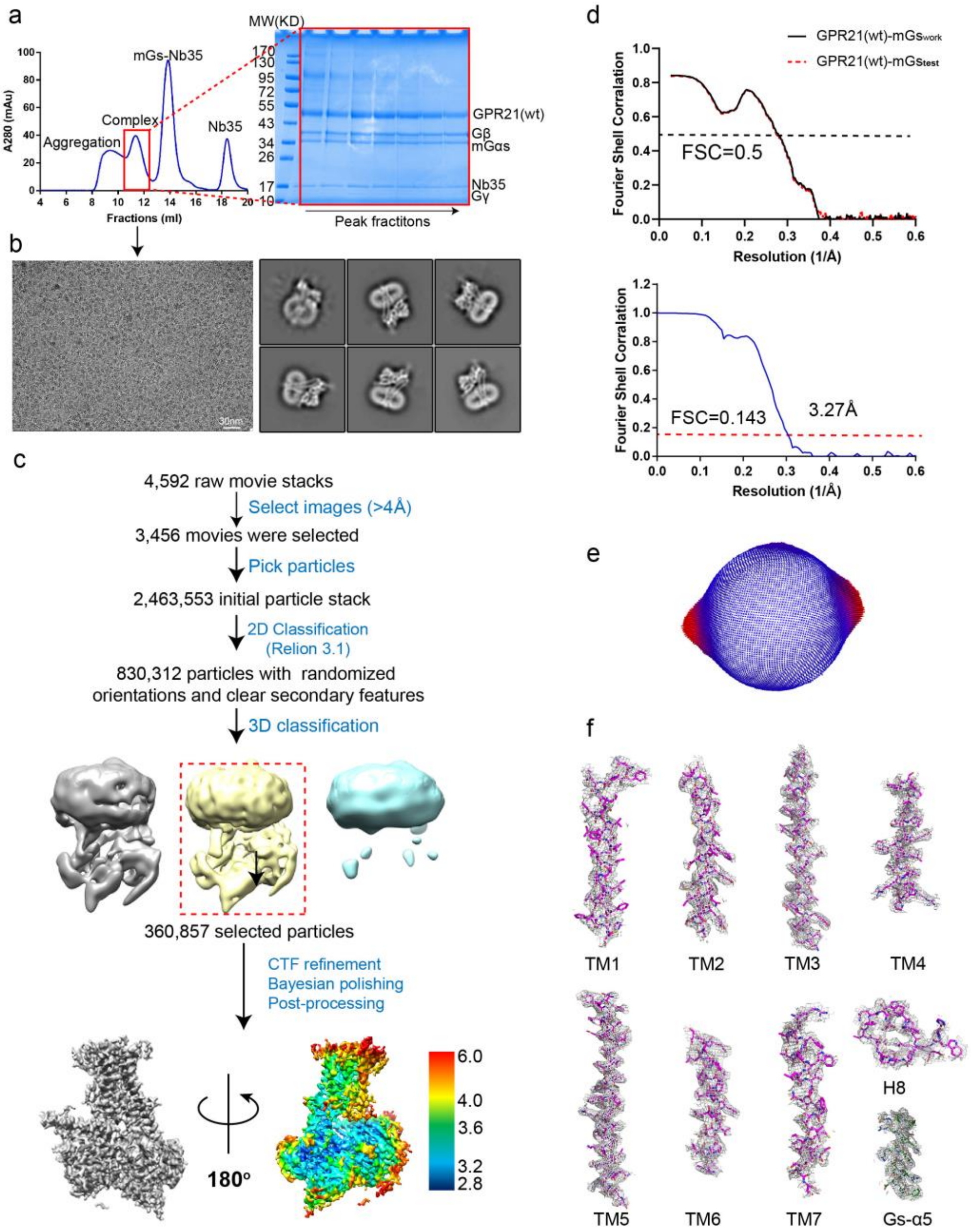

Supplementary Figure 1 | Cryo-EM sample preparation and analysis for GPR21(wt)–mGs. **a,** Left panel, analytical size-exclusion chromatography (SEC) of the purified GPR21(wt)-mGs protein complex, and, right panel, SDS–PAGE analyses of complex after size exclusion. wt, wild type. Experiments were repeated three times with similar results. **b,** Representative cryo-EM micrograph and reference-free two-dimensional class averages of the GPR21(wt)–mGs complex. **c,** Workflow of cryo-EM data processing for GPR21(wt)–mGs complex. **d,** Gold-standard FSC curves of the GPR21(wt)–mGs complex, indicating the resolution are 3.27 Å at the FSC = 0.143. **e,** Angular distribution of the particles used for the final reconstruction of the GPR21(wt)–mGs complex. **f,** Cryo-EM density map and GPR21(wt)-mGs complex model are shown for all transmembrane helices, helix 8 (H8), ECL2 of GPR21, and α5 in the mGαs protein.

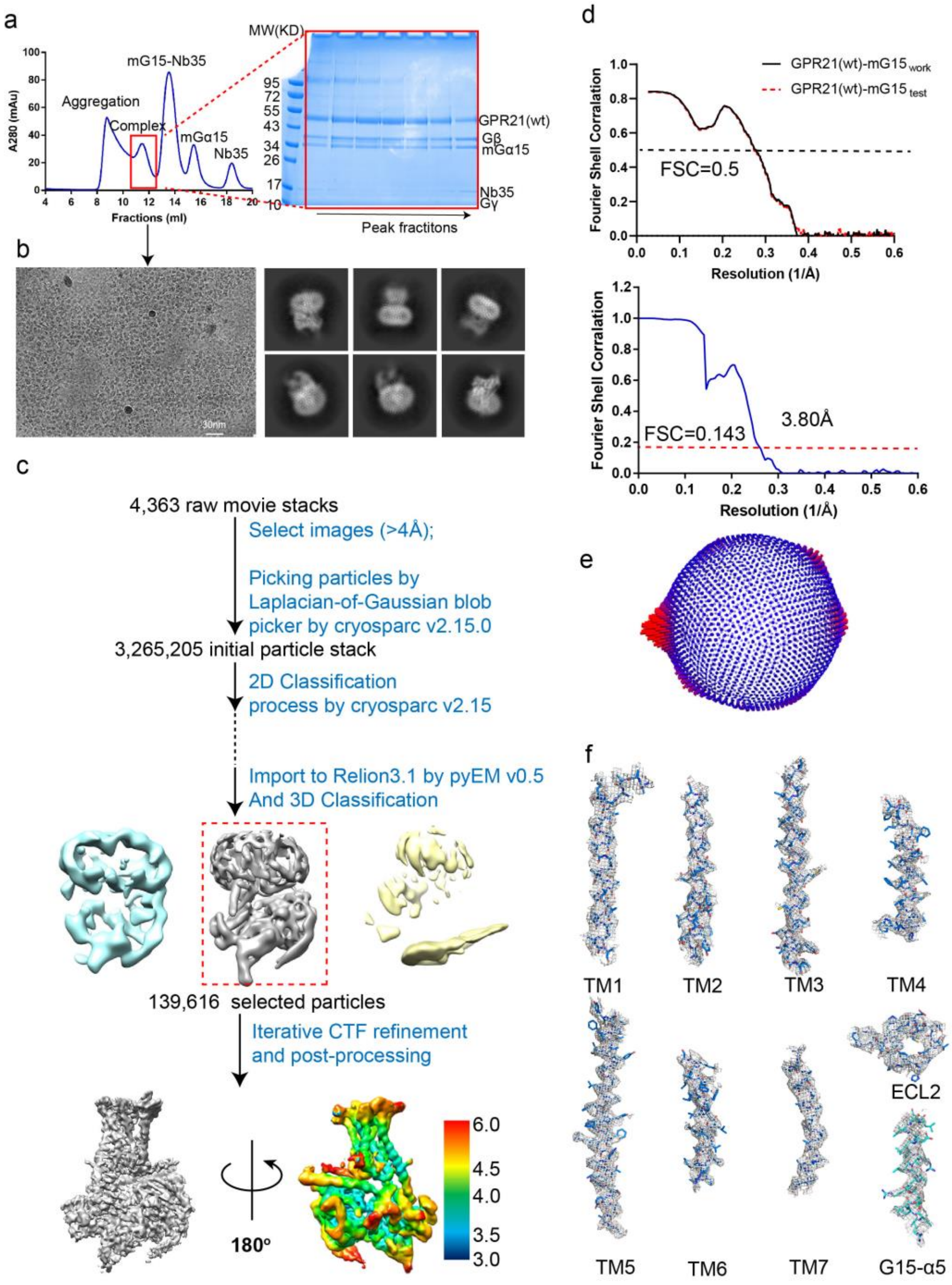

Supplementary Figure 2 | Cryo-EM sample preparation and analysis for GPR21(wt)–mG15. a, Left panel, analytical size-exclusion chromatography (SEC) of the purified GPR21(wt)-mG15 protein complex, and, right panel, SDS–PAGE analyses of complex after size exclusion. wt, wild type. Experiments were repeated three times with similar results. **b,** Representative cryo-EM micrograph and reference-free two-dimensional class averages of the GPR21(wt)–mG15 complex. **c,** Workflow of cryo-EM data processing for GPR21(wt)–mG15 complex. **d,** Gold-standard FSC curves of the GPR21(wt)–mG15 complex, indicating the resolution are 3.80 Å at the FSC = 0.143. **e,** Angular distribution of the particles used for the final reconstruction of the GPR21(wt)–mG15 complex. **f,** Cryo-EM density map and GPR21(wt)-mG15 complex model are shown for all transmembrane helices, helix 8 (H8), ECL2 of GPR21, and α5 in the mGα15 protein.

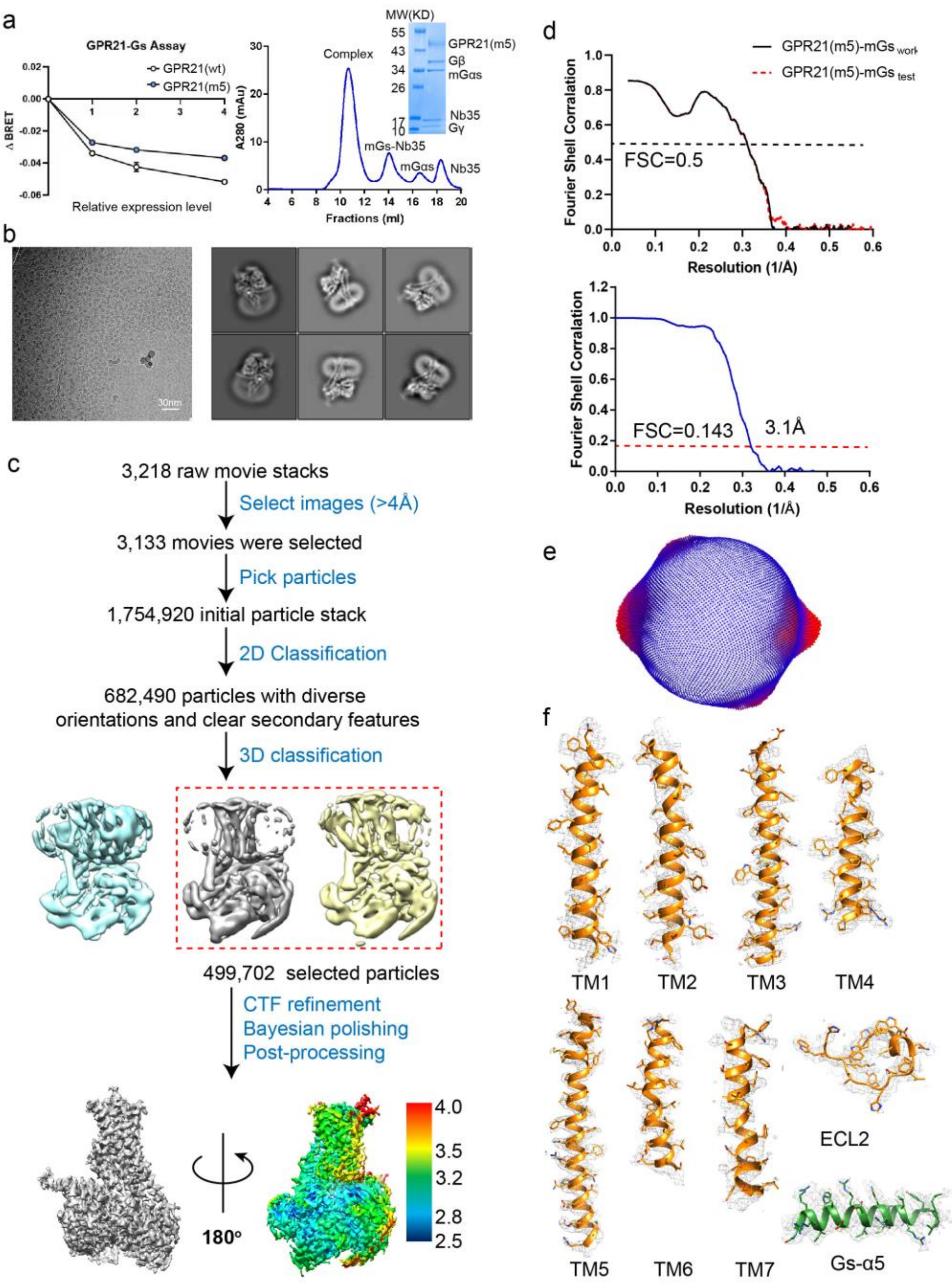

Supplementary Figure 3 | Cryo-EM sample preparation and analysis for GPR21(m5)–mGs. a, Left panel, Constitutive activities of GPR21 receptor in Gs. The gradient cell surface expression levels of GPR21 receptor were achieved by adjusting the transfecting amounts of plasmids encoding the respective receptor in HEK293 cells. Data were from three independent experiments. ΔBRET: the change of bioluminescence resonance energy transfer value. Right panel, SEC and SDS–PAGE analyses of complex after size exclusion. m5, 5mutations. Experiments were repeated three times with similar results. **b,** Representative cryo-EM micrograph and reference-free two-dimensional class averages of the GPR21(m5)–mGs complex. **c,** Workflow of cryo-EM data processing for GPR21(m5)–mGs complex. **d,** Gold-standard FSC curves of the GPR21(m5)–mGs complex, indicating the resolution are 3.1 Å at the FSC = 0.143. **e,** Angular distribution of the particles used for the final reconstruction of the GPR21(m5)–mGs complex. **f,** Cryo-EM density map and GPR21(m5)-mGs complex model are shown for all transmembrane helices, helix 8 (H8), ECL2 of GPR21, and α5 in the mGαs protein.

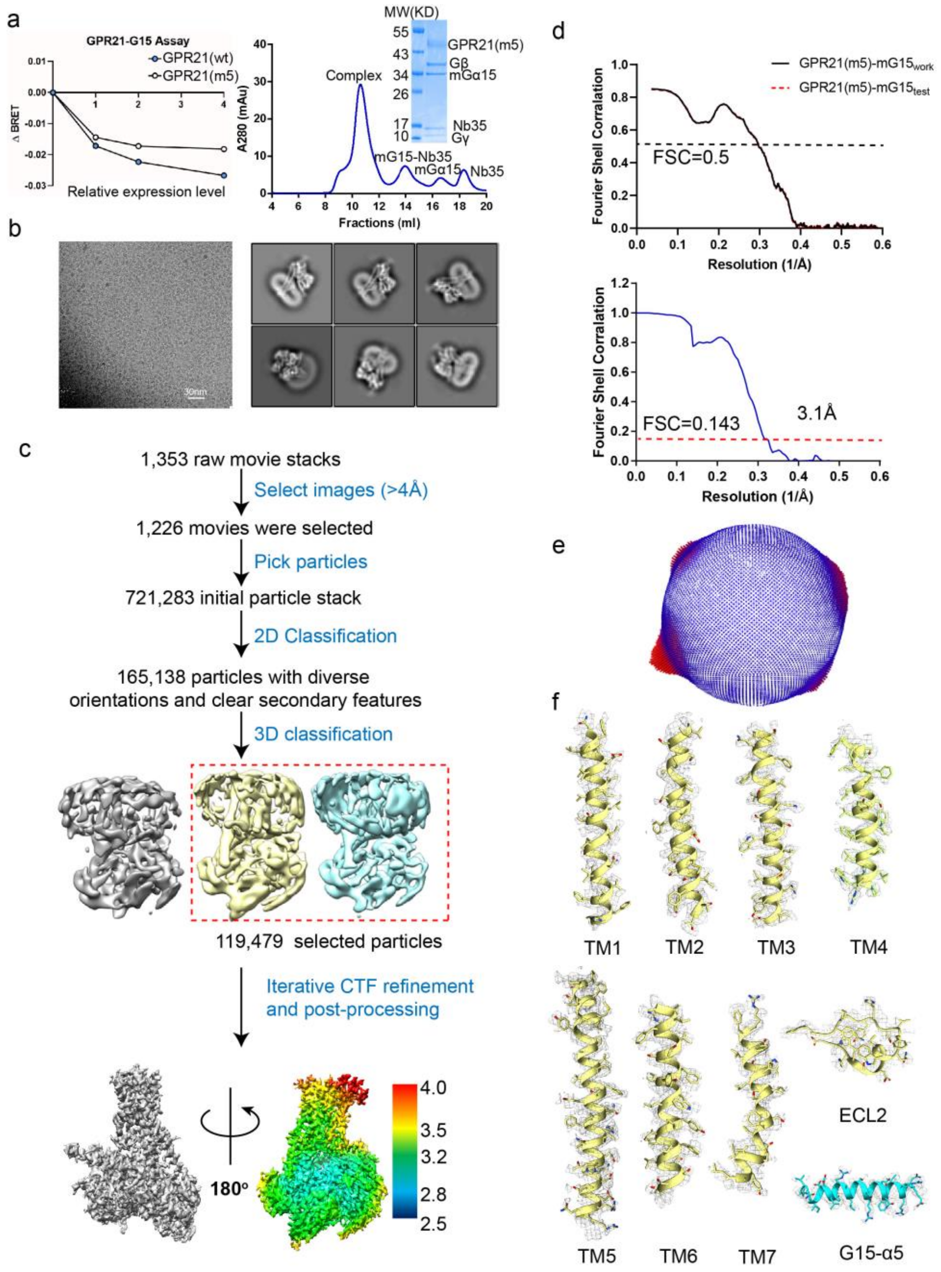

Supplementary Figure 4 | Cryo-EM sample preparation and analysis for GPR21(m5)–mG15. a, Left panel, Constitutive activities of GPR21 receptor in G15. The gradient cell surface expression levels of GPR21 receptor were achieved by adjusting the transfecting amounts of plasmids encoding the respective receptor in HEK293 cells. Data were from three independent experiments. ΔBRET: the change of bioluminescence resonance energy transfer value. Right panel, SEC and SDS–PAGE analyses of complex after size exclusion. m5, 5mutations. Experiments were repeated three times with similar results. **b,** Representative cryo-EM micrograph and reference-free two-dimensional class averages of the GPR21(m5)–mG15 complex. **c,** Workflow of cryo-EM data processing for GPR21(m5)–mG15 complex. **d,** Gold-standard FSC curves of the GPR21(m5)–mG15 complex, indicating the resolution are 3.1 Å at the FSC = 0.143. **e,** Angular distribution of the particles used for the final reconstruction of the GPR21(m5)–mG15 complex. **f,** Cryo-EM map and GPR21(m5)-mG15 model are shown for all transmembrane helices, helix 8 (H8), ECL2 of GPR21, and α5 in the mGα15 protein.

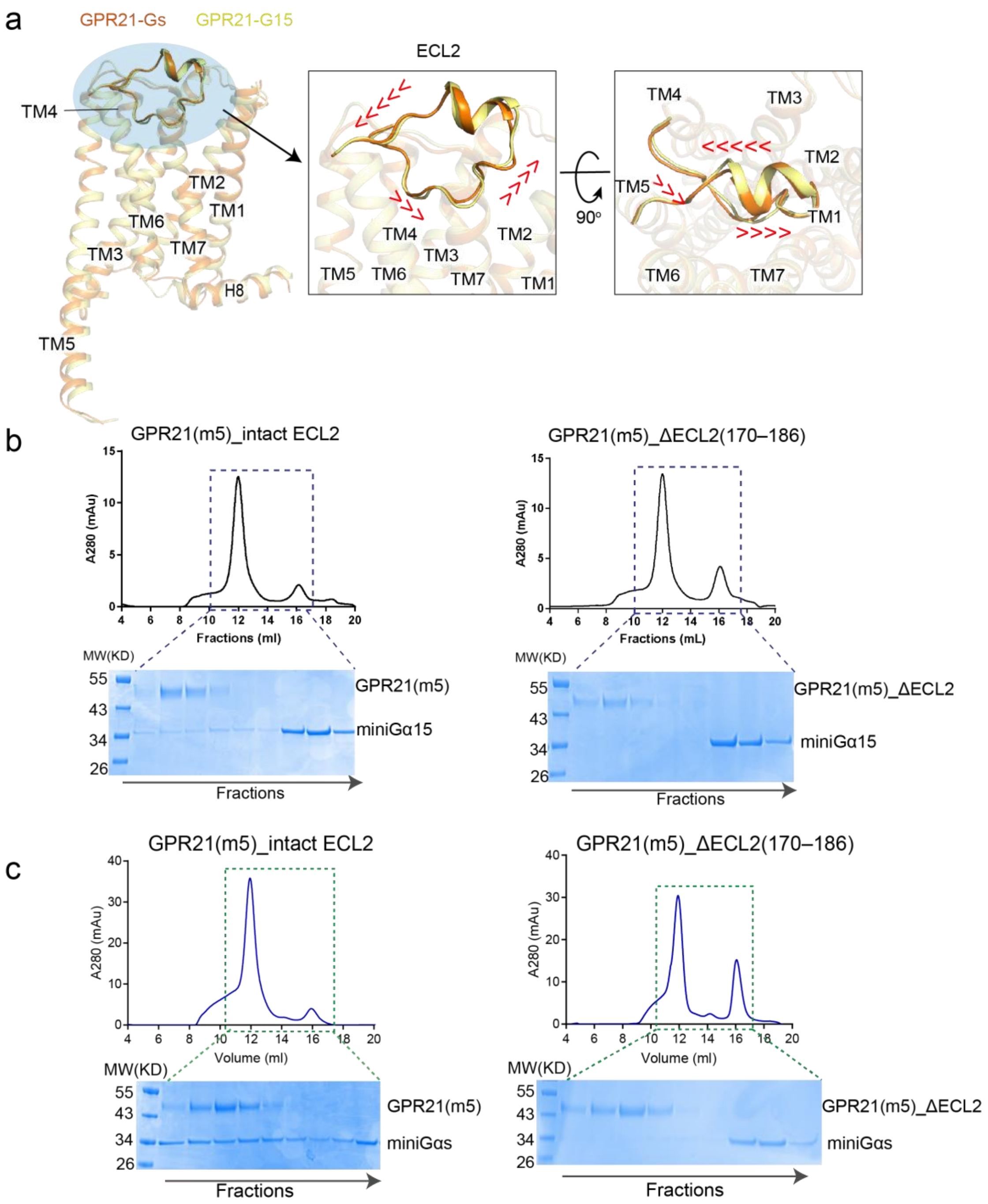

Supplementary Figure 5 | Analysis of ECL2 in GPR21-Gs and GPR21-G15. a, Overall structural comparison of GPR21-Gs and GPR21-G15. ECL2 are shown in magnified view. **b, c,** Analytical SEC (top) and SDS-PAGE gels (bottom) of miniGα protein bound to purified GPR21(m5). Experiments were repeated three times with similar results. Left panel, miniGα protein forms a complex with purified GPR21(m5) containing intact ECL2; Right panel, miniGα protein cannot form a complex with purified GPR21(m5)_ΔECL2 (deleting entire ECL2 residues).

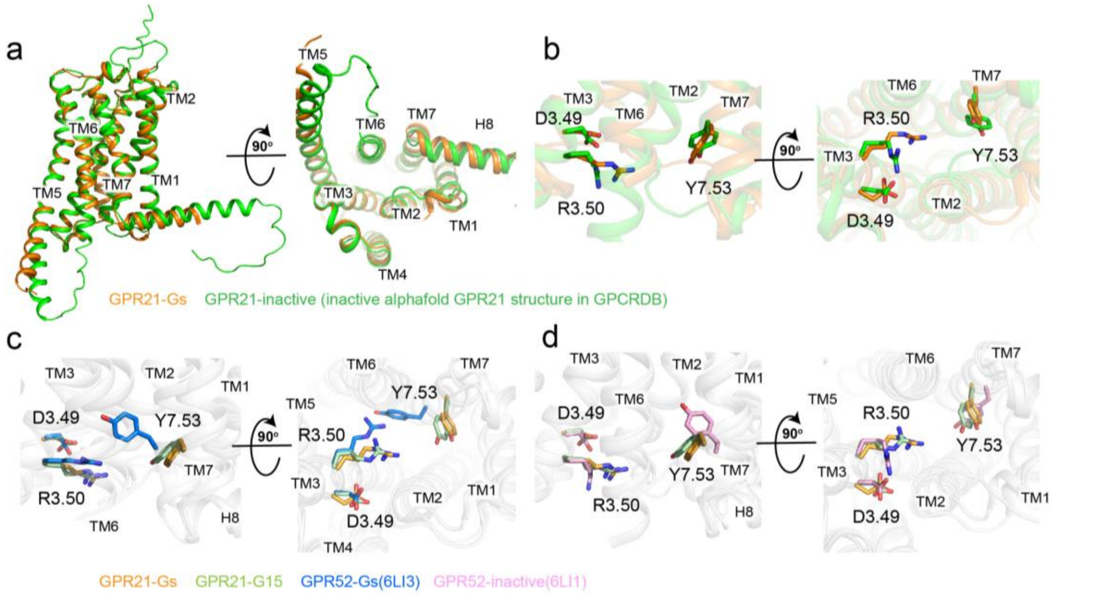

Supplementary Figure 6 | G protein-bound conformation of GPR21 in comparison to GPR52 and alphafold-predicted inactive GPR21. a, Side and intracellular view of the overlay between GPR21-Gs (orange, cryo-EM) and inactive GPR21 (green, inactive GPR21 predicted by alphafold in GPCRDB) structures. **b,** Side and intracellular view of the comparison at R3.50 and Y7.53 between active (cryo-EM) and inactive (predicted) GPR21. D3.49, R3.50, and Y7.53 are shown as sticks. **c,** Side and intracellular view of the comparison at R3.50 and Y7.53 between GPR21 and active-state GPR52 (PDB: 6LI3). D3.49, R3.50, and Y7.53 are shown as sticks. **d,** Side and intracellular view of the comparison at R3.50 and Y7.53 between GPR21 and Gs-free GPR52 (PDB: 6LI1). D3.49, R3.50, and Y7.53 are shown as sticks.

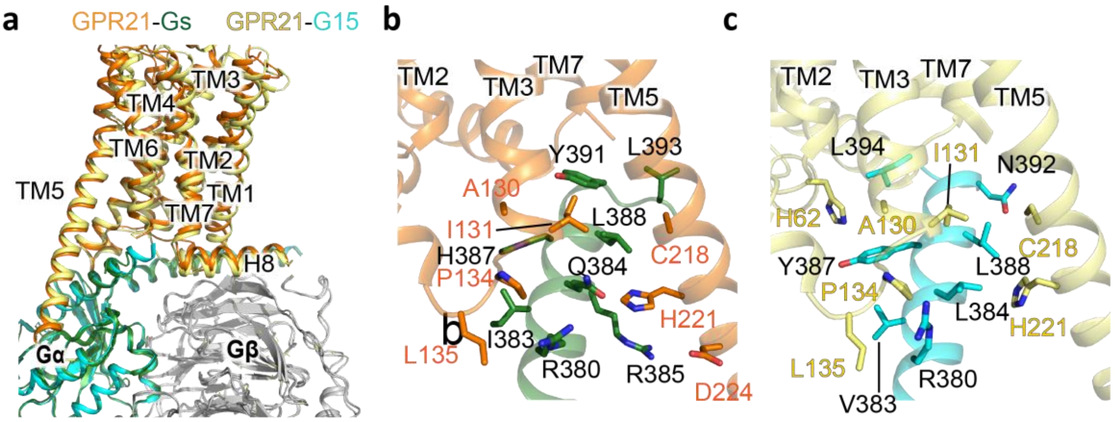

Supplementary Figure 7 | G Protein binding interface in GPR21-Gs and GPR21-G15 complexes. a, Side view of the overlay between GPR21-Gs (orange-green) and GPR21-G15 (yellow-cyan) structures. **b, c** Interface between the α5-helix in Gαs with GPR21 (**b**) and interface between the α5-helix in Gα15 with GPR21 (**c**).

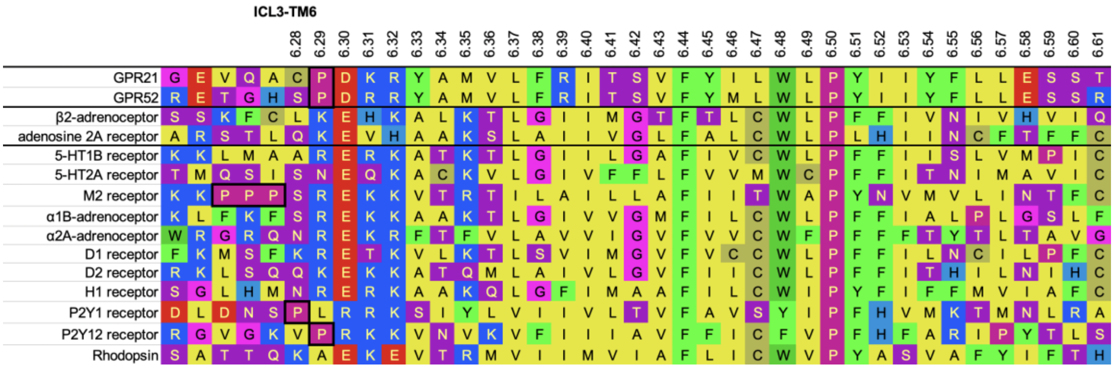

Supplementary Figure 8 | Sequence alignment of GPR21, GPR52 with representative class-A GPCRs at ICL3-TM6 region. Sequence alignment of ICL3-TM6 among GPR21, GPR52, β2-adrenoceptor, adenosine 2A receptor, 5-HT1B receptor, 5-HT2A receptor, M2 receptor, α1B-adrenoceptor, α2A-adrenoceptor, D1 receptor, D2 receptor, H1 receptor and Rhodopsin.

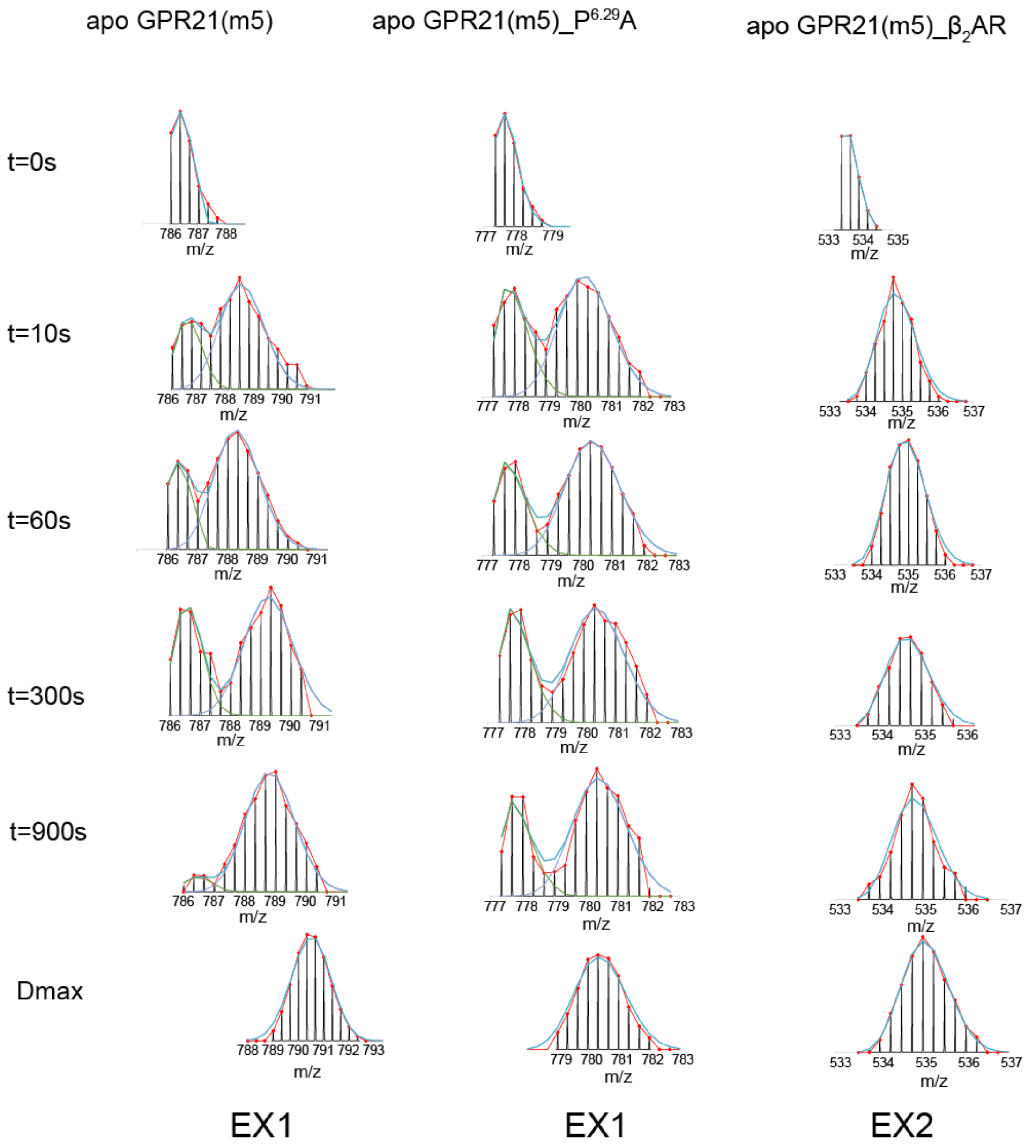

Supplementary Figure 9 | HDX dynamics of apo receptors (GPR21(m5), GPR21(m5)-P$^{6.29}$A and GPR21(m5)-β_2AR). MS spectra of: residues S233^{ICL3}-L254$^{6.37}$ of apo GPR21(m5); P245$^{6.29}$A mutated S233^{ICL3}-L254$^{6.37}$ of apo GPR21(m5)-P$^{6.29}$A; and corresponding peptide in apo GPR21(m5)-β_2AR at t0s, t10s, t60s, t300s, t900s, and a fully deuterated control. Residues S233^{ICL3}-L254$^{6.37}$ of GPR21(m5), P245$^{6.29}$A mutated S233^{ICL3}-L254$^{6.37}$ of GPR21(m5)-P$^{6.29}$A display EX1 exchange pattern whereas corresponding peptide in GPR21(m5)-β_2AR shows EX2 kinetics. The bimodal distribution following EX1 kinetics is further deconvoluted by HX express2 software.

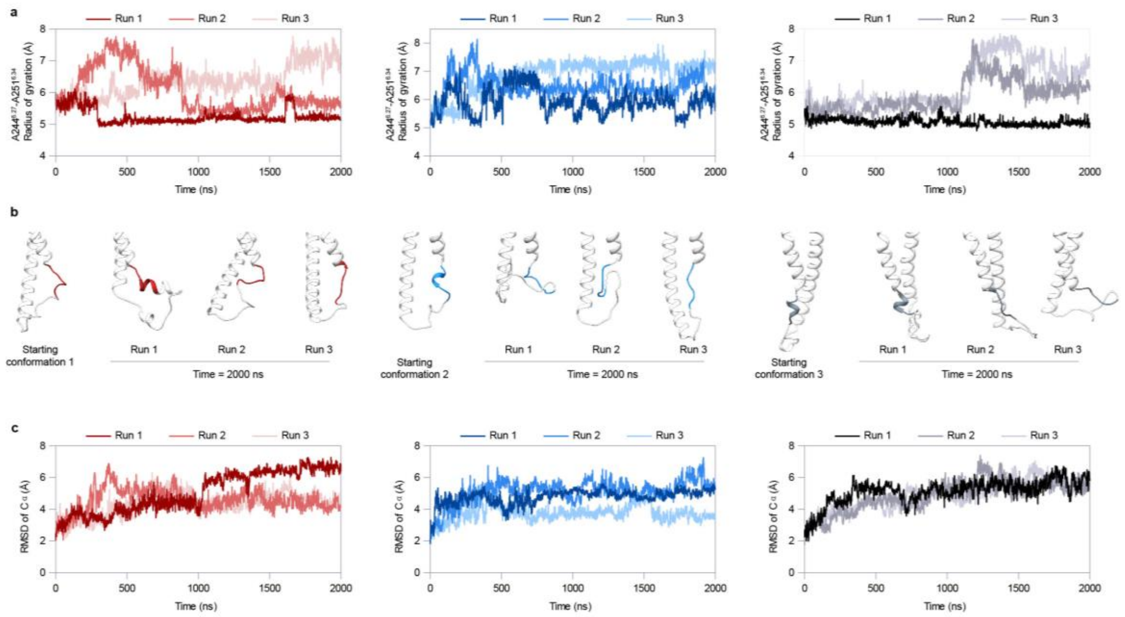

Supplementary Figure 10 | MD simulations of GPR21. a, Radius of gyration of GPR21 TM6 cytoplasmic end (A244$^{6.27}$-A251$^{6.34}$) during 2 μs MD simulations. **b,** Conformations of ICL3-TM6 cytoplasmic end at starting point and end of simulations. **c,** Root-mean-square deviations (RMSD) of Cα atoms of GPR21 during MD simulations.

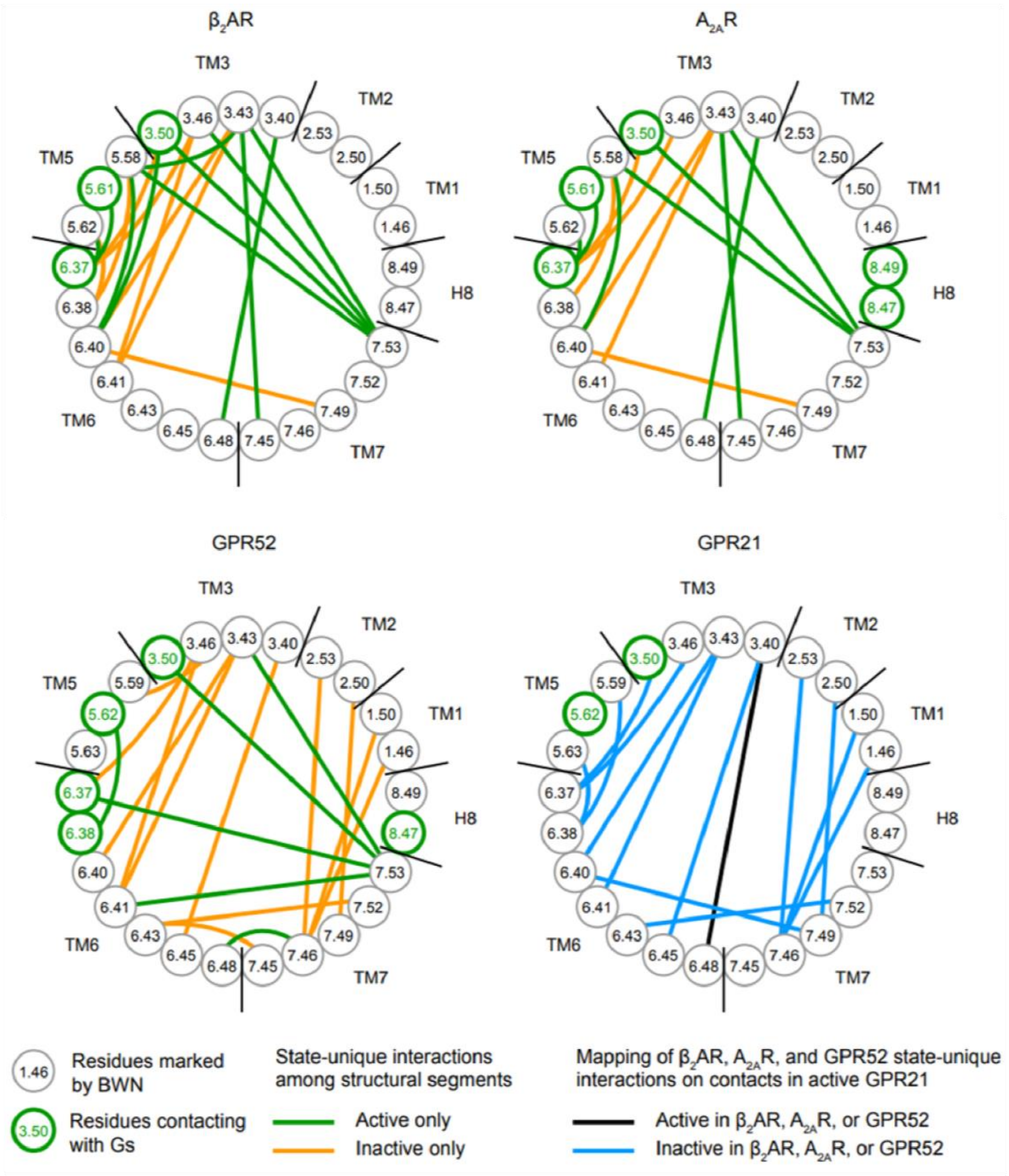

Supplementary Figure 11 | Inter-helical interactions of β_2AR, A_{2A}R, GPR21 and GPR52. Active/inactive state-unique inter-helical interactions (see Methods) in β_2AR/A_{2A}R/GPR52 and mapping in the Gs-bound GPR21 structure.

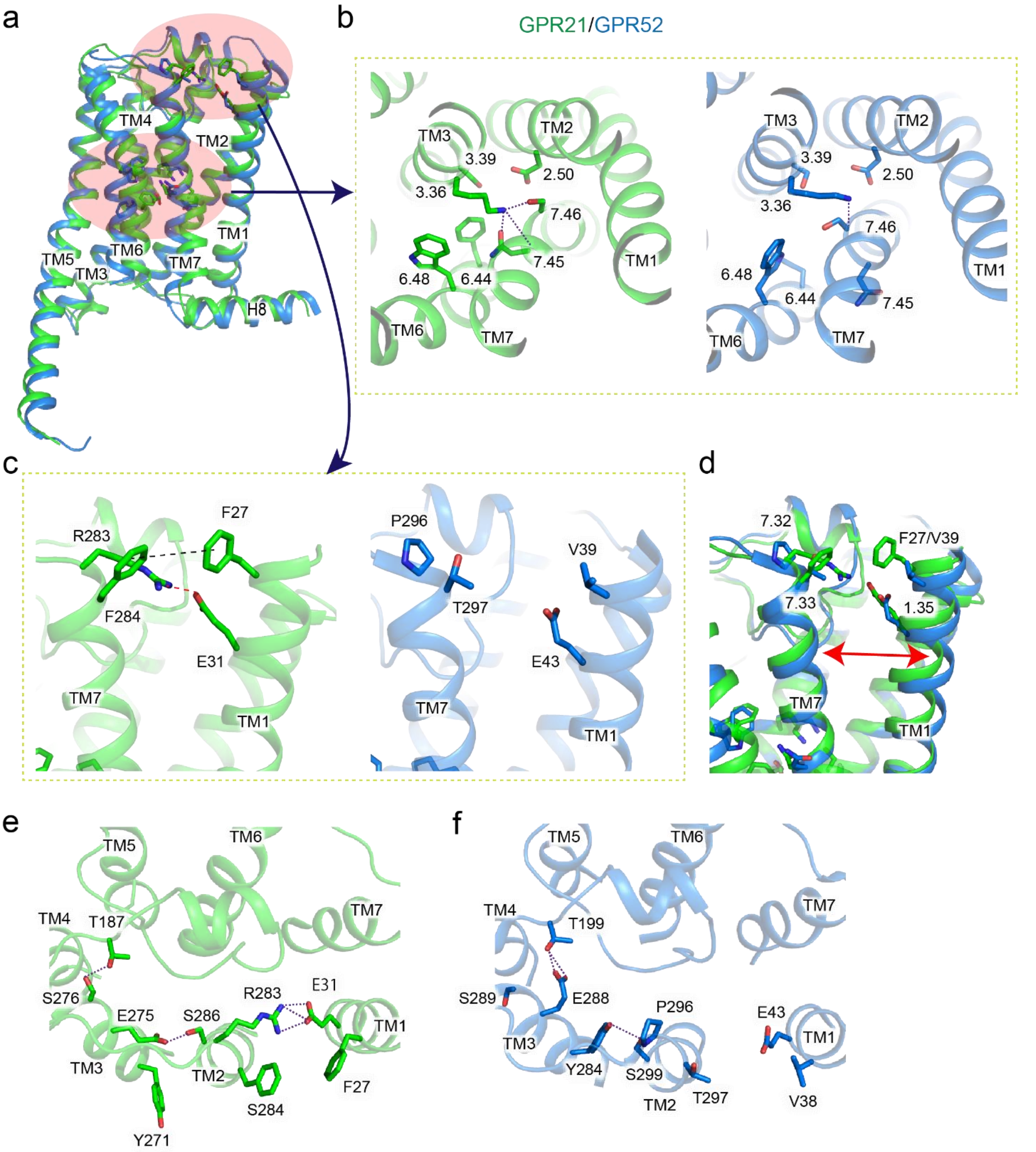

Supplementary Figure 12| Comparisons of GPR21/GPR52 active-state conformation and proposed key residues. a, 7TM comparison of GPR21-Gs (green) and GPR52-Gs (blue). Colour key is used throughout this figure. **b,** Key residues around residue-3.36 in GPR21 and GPR52. The key interactions are shown as purple dashed lines. **c, d** Side view (**c**) and comparison (**d**) of GPR21 and GPR52. **e, f** Top view of GPR21 (**e**) and GPR52 (**f**). Key residues are shown as sticks.

Supplementary Table 1. Cryo-EM data collection, refinement and validation statistics

	GPR21(wt)-Gs	GPR21(wt)-G15	GPR21(m5)-Gs	GPR21(m5)-G15
PDB ID	8HJ1	8HJ2	8HIX	8HJ0
**Data collection and processing**				
Magnification	105,000	105,000	130,000	130,000
Voltage (kV)	300	300	300	300
Electron exposure (e-/Å^2)	60	60	60	60
Defocus range (μm)	-0.7 to -2.2	-0.7 to -2.2	-0.7 to -2.2	-0.7 to -2.2
Pixel Size (Å)	0.832	0.832	1.04	1.04
Symmetry imposed	C1	C1	C1	C1
Initial particle images (no.)	2,463,553	3,265,205	1,754,920	721,283
Final particle images (no.)	360,857	139,616	499,702	119,479
Map resolution (Å)	3.3	3.8	3.1	3.1
FSC threshold	0.143	0.143	0.143	0.143
Map resolution range (Å)	2.8 ~ 9.52	3.25 ~ 7.83	2.44 ~ 6.71	2.77 ~ 6.03
**Refinement**				
**Model vs Map Resolution (FSC=0.5)**	3.5	3.8	3.2	3.2
Map sharpening B factor (Å^2)	-100	-100	-103.745	-90.2788
Model composition				
Non-hydrogen atoms	8263	7467	8274	8260
Protein residues	1043	1027	1043	1043
Ligands	0	0	0	0
B factors (Å^2)				
protein	87.97	104.54	44.15	52.95
Ligand	N/a	N/a	N/a	N/a
R.m.s. deviations				
Bond lengths (Å)	0.006	0.007	0.006	0.006
Bond angles (°)	0.985	1.199	0.937	1.065
Validation				
MolProbity score	1.71	1.65	1.65	1.67
Clash score	6.95	5.66	6.64	6.35
Poor rotamers (%)	0.00	0.00	0.00	0.00
Ramachandran plot				
Favored (%)	95.53	95.06	95.92	95.43
Allowed (%)	4.67	4.94	4.08	4.57
Disallowed (%)	0.00	0.00	0.00	0.00

Supplementary Table 2. Basal activity of wild-type GPR21 and ECL2 mutants, measured by BRET assays

Constructs	Gs activation (ΔBRET)	P value	G15 activation (ΔBRET)	P value
WT GPR21	-0.0503±0.0019		-0.0150±0.0003	
GPR21_C181A	-0.0121±0.0005***	0.0007	-0.0045±0.0002***	0.0009
GPR21_K170E	-0.0131±0.0003***	0.0009	-0.0065±0.0001***	0.0009
GPR21_Δ170-178(GS)	-0.0127±0.0008***	0.0006	-0.0044±0.0002***	0.0005
GPR21_Δ170-186	-0.0038±0.0002***	0.0010	-0.0020±0.0001***	0.0002
GPR21_Δ178-186(GS)	-0.0051±0.0003***	0.0007	-0.0031±0.0002***	0.0004

Data are mean ± s.e.m. from at least three independent experiments. ***$P < 0.001$ by two-way analysis of variance (ANOVA) without repeated measures followed by Dunnett's post hoc test compared to the response of wild type.

Supplementary Table 3. Functional assay of ligand binding site mutations on GPR21, measured by cAMP accumulation assays (HTRF)

Constructs	DMSO-induced activation (response%)	c17-induced activation (response%)	P value	7m-induced activation (response%)	P value
WT GPR21	99.92±5.09	102.28±1.95 n.s.	0.1728	101.38±6.66 n.s.	0.4314
GPR21_N-term GPR52	83.38±8.74	122.84±2.35***	P <0.0001	112.82±3.57***	P <0.0001
GPR21_N-term mutations	65.61±6.03	96.89±5.00***	P <0.0001	97.95±2.63***	P <0.0001
GPR21_7TM mutations	74.25±9.40	84.21±5.13 n.s.	0.5721	74.30±8.41 n.s.	0.3691
GPR21_ N-term mutations (P16L)	74.11±4.86	75.21±4.49 n.s.	0.6283	74.21±3.02 n.s.	0.9679
GPR21_ N-term mutations (Y22L)	65.55±6.33	83.76±4.10***	P <0.0001	85.25±6.36***	0.0008
GPR21_ N-term mutations (S23E)	63.57±5.21	88.03±2.77***	P <0.0001	83.22±5.01***	P <0.0001
GPR21_ N-term mutations (D26N)	85.00±6.05	94.04±2.49**	0.0020	92.37±2.26*	0.0195
GPR21_ N-term mutations (V27F)	89.04±5.57	102.18±3.41***	0.0003	97.93±4.10**	0.0047
GPR21_ N-term mutations (T32V)	95.95±1.23	102,18±3.41***	P <0.0001	100.37±4.91 n.s.	0.0853
HEK293T	1.26±9.86	0.35±14.64 n.s.	0.8607	-1.61±11.79 n.s.	0.6992

Data are mean ± s.e.m. from at least three independent experiments. n.s., not significance, ***P < 0.001, **P < 0.01, *P < 0.05 by two-way analysis of variance (ANOVA) without repeated measures, followed by Dunnett's post hoc test. The effects of receptor on the ligand binding of c17 and 7m compared to DMSO-response.

Supplementary Table 4. Basal activity of wild-type GPR21 and GPR21 chimeras, measured by BRET assays

Constructs	Gs activation of GPR21 (ΔBRET)	P value
WT GPR21	-0.0503±0.0018	
GPR21_A_{2A}R (chimera)	-0.0248±0.0012***	0.0005
GPR21_β_2AR (chimera)	-0.0175±0.0004***	0.0009
GPR21_P6.29A	-0.0244±0.0005***	0.0010

Data are mean ± s.e.m. from at least three independent experiments. ***$P < 0.001$ by two-way analysis of variance (ANOVA) without repeated measures followed by Dunnett's post hoc test compared to the response of wild type.

Supplementary Table 5. Relative ratio between the two populations (Left and Right) for apo-GPR21 (m5) and apo-GPR21(m5)_$P^{6.29}$A

**apo GPR21 (m5)**	10s	60s	300s	900s
bimodal(left) population %	26.14±2.3	26.14±2.3	32.69±1.88	8.92±2.26
bimodal(right) population %	74.46±4.06	73.86±2.3	67.31±1.88	91.08±2.26
**apo GPR21(m5)_$P^{6.29}$A**	10s	60s	300s	900s
bimodal (left) population %	33.15±2.74	31.99±2.16	32.75±1.64	34.09±3.59
bimodal (right) population %	66.85±2.74	68.01±2.16	67.25±1.64	65.91±3.59

Supplementary Table 6. Centroid value summery

apo GPR21 (m5)	10s	60s	300s	900s	Dmax
mean peak centroid	787.47±0.03	787.40±0.07	787.68±0.15	787.85±0.02	789.05±0.06
left peak centroid	786.58±0.01	786.50±0.03	786.68±0.07	786.66±0.10	NA
right peak centroid	787.74±0.05	787.75±0.09	788.24±0.10	787.97±0.06	NA
apo GPR21(m5)_P6.29A	10s	60s	300s	900s	Dmax
mean peak centroid	778.43±0.02	778.48±0.02	778.49±0.01	778.42±0.06	778.77±0.01
left peak centroid	777.89±0.01	777.89±0.02	777.87±0.01	777.88±0.01	NA
right peak centroid	778.72±0.03	778.75±0.01	778.75±0.01	778.71±0.03	NA

Supplementary Table 7. HDX-MS summary

Data Set	GPR21(m5)	GPR21(m5)_P6.29A	GPR21(m5)_β2AR
HDX reaction details	50 mM HEPES, 50 mM NaCl, 2 mM DTT, pD_{read} =7.9, 4 °C	50 mM HEPES, 50 mM NaCl, 2 mM DTT, pD_{read} =7.9, 4 °C	50 mM HEPES, 50 mM NaCl, 2 mM DTT, pD_{read} =7.9, 4 °C
HDX time course (min)	0.17,1,5,15	0.17,1,5,15	0.17,1,5,15
HDX control samples	Maximally-labeled control (WT protein)	Maximally-labeled control (WT protein)	Maximally-labeled control (WT protein)
Back-exchange (mean / IQR)	37.92%/ 29.30%	30.50%/30.1%	42.95%/37.73%
# of Peptides	467	467	493
Sequence coverage	44%	44%	49%
Average peptide length / Redundancy	16.95 / 39	19.59 / 63	16.59 / 64
Replicates (biological or technical)	3 (technical)	3 (technical)	3 (technical)
Repeatability	0.050 (average standard deviation)	0.0461 (average standard deviation)	0.0286 (average standard deviation)
